# Reduced dispersibility of flushable wet wipes after wet storage

**DOI:** 10.1038/s41598-021-86971-z

**Published:** 2021-04-12

**Authors:** Thomas Harter, Ingo Bernt, Stefanie Winkler, Ulrich Hirn

**Affiliations:** 1 Institute of Bioproducts and Paper Technology, Graz University of Technology, Inffeldgasse 23, 8010 Graz, Austria; 2 Kelheim Fibres GmbH, Regensburger Straße 109, Kelheim, Germany; 3 CD Laboratory for Fibre Swelling and Paper Performance, Inffeldgasse 23, 8010 Graz, Austria

**Keywords:** Chemical engineering, Pollution remediation, Polymers

## Abstract

Scientific publications and newsfeeds recently focused on flushable wet wipes and their role in sewage system blockages. It is stated that although products are marked as flushable, they do not disintegrate after being disposed of via the toilet. In this work it is shown that wetlaid hydroentangled wet wipes lose their initially good dispersive properties during their storage in wet condition. As a consequence, we are suggesting to add tests after defined times of wet storage when assessing the flushability of wet wipes. Loss of dispersibility is found for both, wet wipes from industrial pilot production and wipes produced on laboratory pilot facilities. We found it quite surprising that the wet wipes’ dispersibility is deteriorating after storage in exactly the same liquid they are dispersed in, i.e. water. This is probably why the effect of wet storage has not been investigated earlier. It is demonstrated that the deteriorating dispersibility of these wipes is linked to the used type of short cellulosic fibres — only wipes containing unbleached softwood pulp as short fibre component were preserving good dispersibility during wet storage. Possible mechanisms that might be responsible are discussed, e.g. long term fiber swelling causing a tightening of the fiber network, or surface interdiffusion.

## Introduction

Wet wipes, with their broad range of applications, are part of the modern life and can be found in many households. Especially the demand on personal hygiene products has increased and is forecasted to rise by 8.0% p.a. in the next years^1^. Convenience and hygiene require the easy disposal of these wipes, preferably right after use. This creates a market for biodegradable and flushable products. Both terms must be discussed separately as wipes that are marked flushable are not necessarily biodegradable. Therefore, the term “truly flushable” was introduced^2^ for wipes that, after successfully being disposed via sewage, are able to disperse and are also able to degrade in nature.

Biodegradability thereby is defined as a breakdown mechanism that creates simple substances in a biological way^3,4^. In the list of fibres used in nonwovens^1^ two groups of polymers, cellulose and PLA (polylactic acid), fulfil this criterion^5^, where the biodegradability of PLA in a marine environment is in discussion recently^6^. Over 50% of the raw materials used in wet wipes are cellulosic natural biopolymers such as regenerated fibres (Rayon and Lyocell), wood pulp and cotton^1^, that are all able to biodegrade in an aquatic environment^7^. For a product to be fully biodegradable it must entirely consist of these materials, however synthetic fibres can be found in wet wipes labelled as flushable^8,9^. Toilet paper as an example is a material which solely consists of cellulosic pulp and disintegrates well after disposal^10^. Wet wipes produced as a blend of viscose fibres and wood pulp show sufficient strength for proper usage of the wipe^11^. They consist only of cellulosic material^11^ and are therefore biodegradable^12^. In these wipes the long viscose fibres form the load-carrying structure providing wet strength and the pulp fibres attached to the body are responsible for liquid absorption and dispersive properties^13^. The production process of these wipes, a combination of wetlaid forming of the fabric and subsequent hydroentanglement with high-pressure water jets^14^ requires no chemical agents as additives or binders. For disposal it has to be possible to flush the wet wipes down the sewage system.

The guidelines of the nonwoven industry associations (INDA in the US and EDANA in Europe) as well as the water research associations providing recommendations on flushability (IWSFG) are both advising to use the slosh box disintegration test^15,16^ for evaluation of flushability, also compare e.g.^17^. As they are not containing any type of binders nonwovens from pure cellulosic fibres seem to be the most promising material to obtain true flushability, also according to slosh box tests^2,18^.

Flushability and labelling wet wipes as flushable have been in public discussion recently^19–21^. One reason for this public interest could be the newsfeeds that reported major blockages in the New York^22^ and London^23^ sewer system. Fatbergs, as these massive blockages are called in the media, consist of undispersed wet wipes and fatty deposits^23^. These deposits form in the sewer system from disposed fats, oils and greases^24,25^. Field measurements in Berlin^26,27^ and Tokyo^28^ as well suggest that blockages are caused by undispersed wet wipes disposed via the toilet^26^. Next to wrongly disposed non-flushable wipes^29^ these investigations also found so-called flushable wipes that did not disperse properly^17,30,31^. In a broad study investigating flushability of consumer products all 23 tested fabrics, including wet wipes, labelled as flushable were in fact found to only dissolve partly in the tests^17^.

A lot of work has been done investigating the dispersive properties of wetlaid hydroentangled wipes^11,32–37^. Wet wipes are facing the problem that they are contributing to sewer blockages which is documented publicly^21,38^ and scientifically^27^ as they do not disperse. In contrast to these occurrences many publications^32–37^ found good dispersible properties for wet wipe materials directly after production prior to applying any liquid to the fabrics. Taking all the presented findings into account it stands to reason that the storage conditions impact the dispersibility of wet wipes leading to our first hypothesis:

1. Storage conditions and storage time are liable for wet wipes to deteriorate their initially good dispersibility.
  Both the good dispersibility of never wetted nonwovens and the missing dispersibility of consumer-sold wet wipes, whether they are marked flushable or not, were carried out by other work groups using different materials. The findings in these publications were always the same therefore, we add three more statements to our working hypothesis:
2. The production process of the precursor material does only impact the level of dispersibility but not the time dependent behaviour.
3. The type of applied liquid is not influencing any time dependent change in dispersibility. All of these effects that occur in wet wipes can also be caused by deionized water.
4. Choosing appropriate fibres as raw material for the wet wipe production will preserve the good dispersibility of the wipes during shelf storage.

In this work we will show that dispersibility of flushable wet wipes, measured with the slosh box disintegration test, can decrease drastically over wet storage time. For this decrease in dispersibility of the wet wipes during wet storage, we coined the term ’dispersibility ageing effect’. We will show that without wet storage the wipes are showing excellent dispersibility, however they are losing these properties within 24 h of wet storage. This loss of dispersibility is demonstrated for wipes from industrial pilot production as well as for wipes produced on laboratory pilot scale. We will also demonstrate that by selecting suitable fibres to produce the wet wipe, it is possible to obtain wipes with little to no dispersibility ageing, thus proving that the widespread wetlaid/hydroentanglement process is suited to manufacture biodegradable and truly flushable wet wipes. Still, stable dispersibility over wet storage was only found for one set of fibres not currently used for commercial products as far as we know, indicating that typical commercially available wet wipes are deteriorating in their dispersibility properties during wet storage in the consumer package, at least in the first 168 hours.

## Materials and methods

### Raw materials

The fibres used for the wet wipes are a blend of viscose fibres with chemical pulp fibres. The viscose fibres were produced in an industrial scale viscose fibre line^39^ at Kelheim Fibres (grade A and B). Viscose fibre A is a flat viscose fibre with a rectangular cross section, a fibre length of 10 mm and a linear mass density (called *titer* in textile engineering) of 2.4 dtex (2.4 g per 10,000 m fibre). The second fibre (viscose fibre B) has a roundish, irregular-shaped cross section and a fibre length of 8 mm, it is a finer fibre with a titer of 0.9 dtex. The fibres consist of cellulose and have a surface finishing with ethoxylated fatty acid according to viscose fibre industrial standards^40^. The chemical pulps are a bleached softwood kraft pulp (SW-BK) and an unbleached softwood kraft pulp (SW-UBK), both commercially available grades from industrial production. The specifications of these pulps are listed in Table 1. The Kappa number, measured according to ISO 302:2004, is indicating the lignin content of the pulps, nearly all the lignin has been removed from the bleached pulp grade.

**Table 1:** Specifications of the used pulp grades.

Type	Length-weighted average fibre length (mm)	Kappa number ISO 302:2004	Wood type
SW-BK	2.368	0.5	North American Softwood (spruce, pine, fir)
SW-UBK	2.453	10.1	European SW (spruce, pine, larch)

All used materials are of cellulosic origin and therefore biodegradable. The blends of these viscose fibres with the bleached pulp are representing standard recipes for flushable wet wipes produced for consumer markets. The unbleached softwood kraft pulp was only used in the laboratory wipes and represents a prototype for possible future applications.

### Wet wipe production—laboratory pilot scale

The laboratory pilot-scale method has been adapted to mimic the industrial pilot production process described below. The production of the tested wipes includes 4 steps. In a first step an inclined wire wetlaid former with a web width of 290 mm was used to form a web where the fibres were primarily aligned in machine direction. Web speed is 4 m/min. Similar to paper production the nonwoven was dried with air at 160 °C and a web draw of 0.1 m/min. These two steps provided a bulky and weak non-woven fabric. For the proper usage of these fabrics as personal care wet wipes hydroentanglement was used to create the required tensile strength. For this procedure the fabric was passing 3 bars of spray nozzles applying water jets to the fabric, created by pressures of 5, 60 and 70 bars. The water jet nozzles were mounted at 40 holes per inch, each hole 0.1 mm in diameter. Web speed is 5 m/min. After hydroentanglement the web was dried on-line at 130 °C. A full list of the produced reel materials can be found in Table 2. Here the abbreviation describes the production way and the used materials. P.AB.20 therefore refers to a wipe that is produced with the laboratory pilot-scale method using viscose fibre A (A) and bleached softwood kraft pulp (B). The relative amount of viscose fibre in this example was 20% and the rest sums up to the amount of pulp. The targeted grammage of the nonwovens was 65 g/m^2^.

**Table 2:** List of produced laboratory pilot-scale nonwovens.

Name	Viscose fibre	Pulp	Mass ratio Viscose/pulp
P.BB.20	Viscose fibre B	Bleached softwood kraft pulp	20:80
P.AU.20	Viscose fibre A	Unbleached softwood kraft pulp	20:80
P.AB.20	Viscose fibre A	Bleached softwood kraft pulp	20:80
P.AB.30	Viscose fibre A	Bleached softwood kraft pulp	30:70
P.BB.30	Viscose fibre B	Bleached softwood kraft pulp	30:70

PW stands for pilot web, VA/VB for viscose type and BSK/UBSK for the pulp grade.

### Wet wipe production—industrial pilot scale

In order to investigate the behaviour of the actual consumer product also an industrial production facility was used to produce non-woven fabrics. Therefore, one of worldwide largest producer for hydroentangled wetlaid nonwovens provided us with the nonwovens listed in Table 3 manufactured in a standard process for consumer wet wipes. Both components, viscose fibres and pulp, were the same products like in the laboratory pilot-scale produced fabrics, but from different production lots. The industrial machinery to produce the fabrics was similar to the pilot-scale method. Web forming was achieved using the wetlaid process to build the initial network of the nonwoven. The hydroentanglement process in the industrial pilot scale is carried out in-line with the web forming. Therefore, these fabrics were only dried once, after the hydroentanglement.

**Table 3:** List of nonwovens from industrial pilot production.

Name	Viscose fibre	Pulp	Mass ratio Viscose/pulp
I.AB.20	Viscose fibre A	Bleached softwood kraft pulp	20:80
I.BB.20	Viscose fibre B	Bleached softwood kraft pulp	20:80
I.ABB.13.7	Viscose fibre A and B	Bleached Softwood KRAFT pulp	13:7:80

IW stands for industrial web, VA/VB for viscose type and BSK for bleached softwood kraft pulp.

### Wet wipe preparation and storage

For the dispersibility measurement single wipes, 125 × 175 mm, were cut from the reel fabric. The grammage for all wipes was 65 g/m^2^, giving a single wipe the weight of 1.4 g. Orientation effects were eliminated as the wipes were always cut in the same direction. For consumer products the wipes were stored in a lotion aimed to improve skin comfort and scent of the product. For storage we used both, deionized water and lotion. The liquid was applied to the cut wipes in a mass ratio of 2:1 (liquid:ambient condition dry mass of wet wipe) and stored in a closed plastic bag. For the measurements with the lotion the web PW1–VB BSK was used. The lotion consists of 95.9 w% of water and chemicals, similar to a widely used patent^41^. Apart from water the lotion contains 1.5% thickener (Propylenglycol), emulgators (0.8% castor oil and 0.45% silicone polyether), 0.5% buffer (citric acid) as well as stabilizers, other emulgators and preservation agents. To avoid bacterial and fungal decay the bags with the wet wipes were stored in a refrigerator at 4 °C. For each measurement point three wipes were stored in one plastic bag.

### Dispersibility measurement—slosh box disintegration test

Many methods exist to determine the dispersive behaviour of wet wipes^11,34,37^, in this work we used the slosh-box-test. As a testing method that is used by the nonwoven industry^15^ and the water service companies^16^ the slosh box test is a widely accepted method to characterize the dispersibility of wet wipes. The slosh box test was also used in other work^17^. Here over 100 materials, that were intended to be flushable, were tested making the slosh box test a reasonable method to determine dispersibility of wet wipes. The test specifications used in this work were, with alterations as listed below, similar as provided by the INDA/EDANA guidelines^15^. As suggested by the International Water Services Flushability Group (IWSFG)^16^, the organization representing the interests of water service providers, the time for dispersion in the slosh box was set to 30 min.

**Figure 1:**
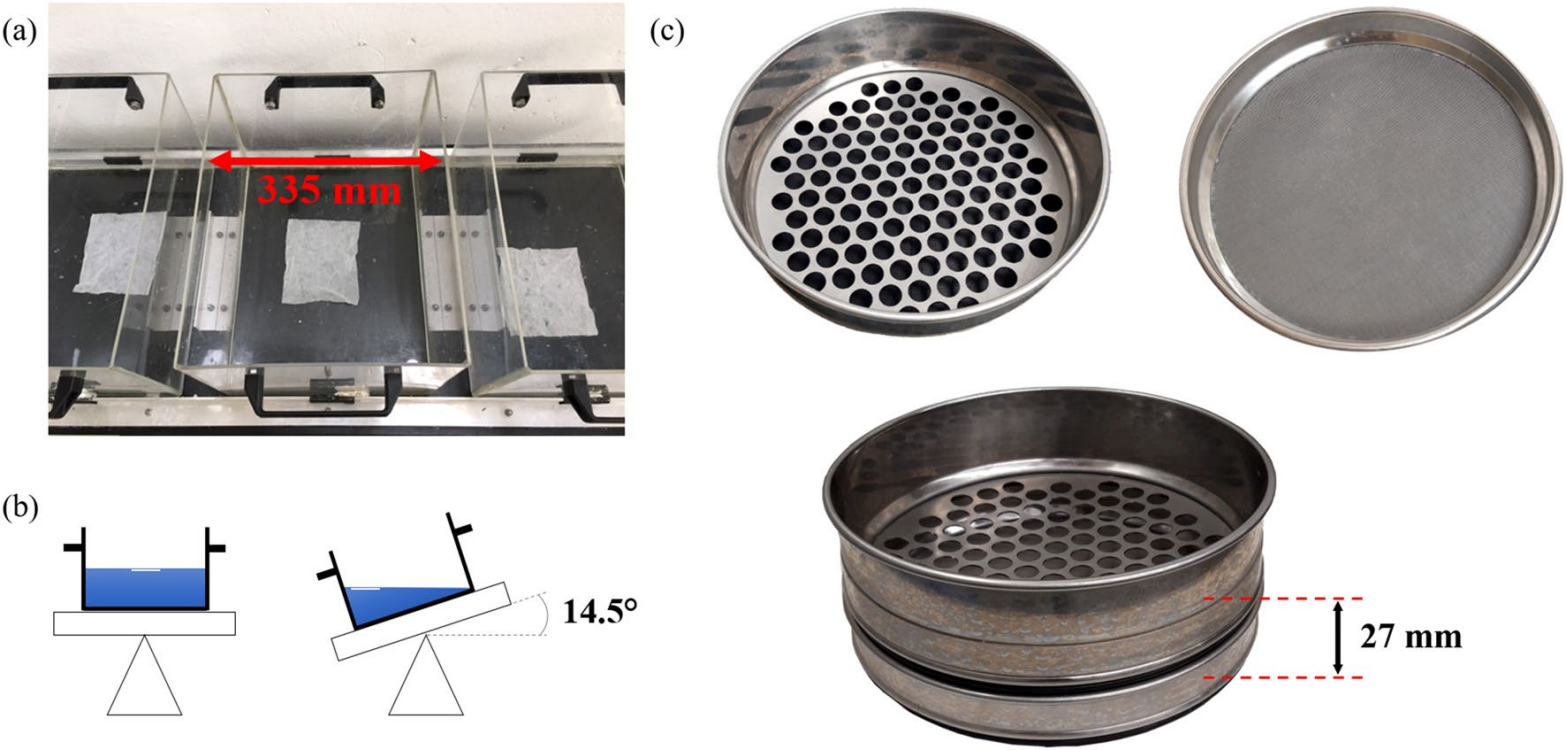
Slosh box principle: (**a**) top view of slosh box with a wipe (125 × 175 mm), (**b**) scheme of slosh box side view with maximum tilting amplitude, (**c**) 12.5 mm hole sieve, 200 µm mesh sieve and both sieves stacked on top of each other with a distance of 27 mm.

For the test a plastic container (435 × 335 × 270 mm) was filled with 2 L of tap water. One wet wipe was placed on the water, as shown in Fig. 1a. An engine was tilting the box back and forth with 26 rpm for 30 min. The maximum tilting angle in both directions is 14.5° (Fig. 1b). The wet wipe was supposed to disintegrate due to the gentle agitation. Subsequently the state of dispersibility was evaluated by collecting the larger non-disintegrated parts of the wipe on a 12.5 mm hole sieve. Therefore the 12.5 mm sieve was stacked on a 200 µm sieve, Fig. 1c, and the suspension was poured within 20 s from a height of 100 mm above the upper sieve. The distance between the two sieves was 27 mm. The fibre material withheld on the sieves was collected and dried at 105 °C for 4 h. Then the mass of the two material fractions from the coarse (*m*_*12.5 mm*_ [g]) and fine (*m*_*200µm*_ [g]) sieve was determined. Dispersibility was calculated via Eq. (1), a higher dispersibility value indicates a better dispersive behaviour.

1 $$Dispersibility= \frac{{m}_{200\upmu m}}{{m}_{200\upmu m} + {m}_{12.5mm}}\left[\mathrm{\%}\right]$$

The used slosh box tester consisted of three liquid containers next to each other (Fig. 1a). Thus, for each measurement point three individual sheets were sloshed, one in each box. After storage the wet wipes were directly put into the slosh box. The wipes were not dried prior to slushing like for other tests^30,42^, as this is not representing actual consumer usage.

## Results and discussion

**Figure 2:**
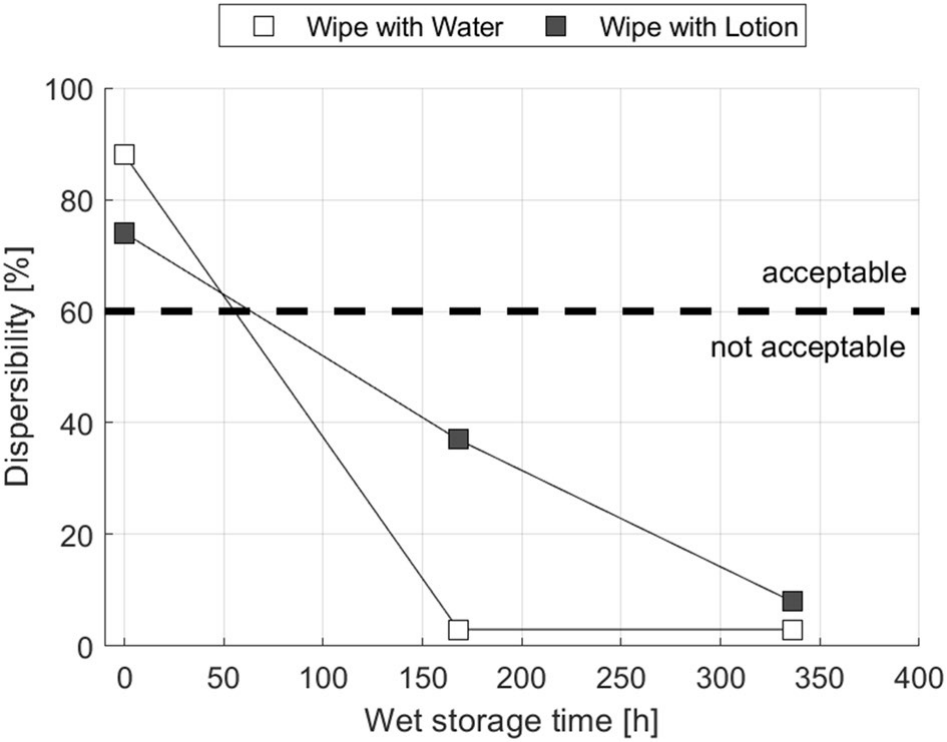
Reduction of wet wipe dispersibility due to wipe storage in water and wet-wipe lotion. Below a value of 60% dispersibility is insufficient according to INDA/EDANA^15^. Sample P.BB.20 made of bleached kraft pulp and round viscose fibre B was used.

Commercial wet wipes are usually treated with lotions improving the customer experience in terms of pleasant smell and skin comfort. The first trial was aimed at comparing if there is a difference in dispersibility loss over time between wet wipes stored in lotion and wet wipes stored in water, as the lotions are consisting mostly of water^41^. Figure 2 represents the difference between a wipe stored in lotion and the same wipe stored in deionized water. Both liquids lead to a pronounced decrease in dispersibility over the wet storage time. It is interesting that water, the liquid used to disperse the wipes, is at the same time the substance causing the solidification of the wipes. While lotion is initially slowing the dispersibility ageing, over longer storage time also the dispersibility for the wet wipes in lotion drops to very low values. This is in full agreement with our hypotheses stating that (1) the wet storage is liable for the deteriorated dispersibility and (3) that the liquid type is not relevant for this effect.

**Figure 3:**
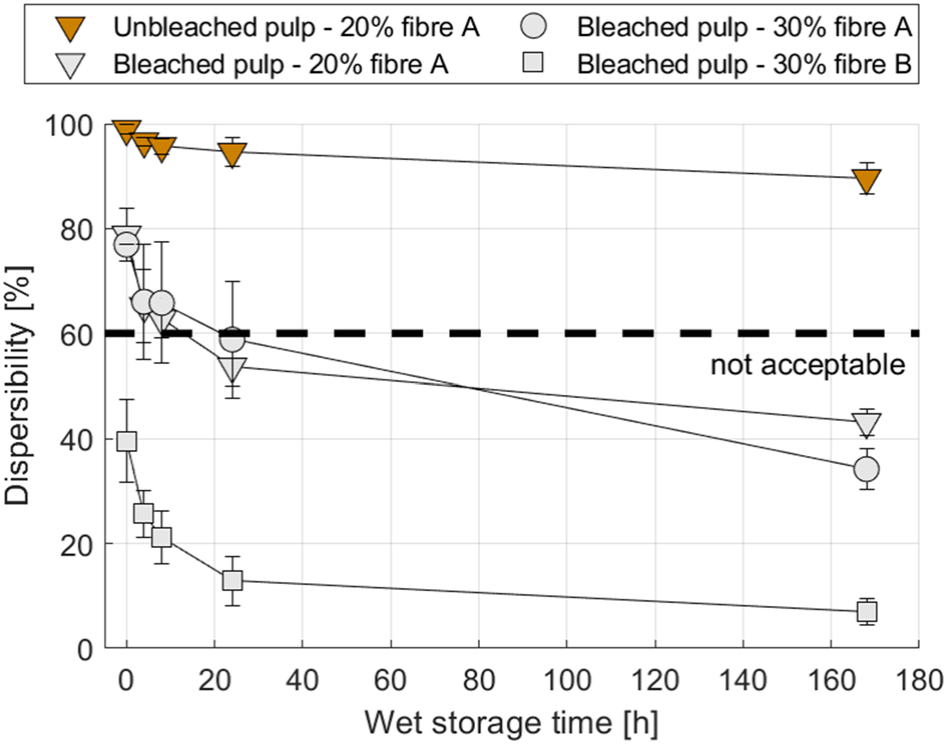
Reduction of wet wipe dispersibility due to storage in water for wipes produced from different raw materials (compare Table 2). Error bars represent one standard deviation.

**Figure 4:**
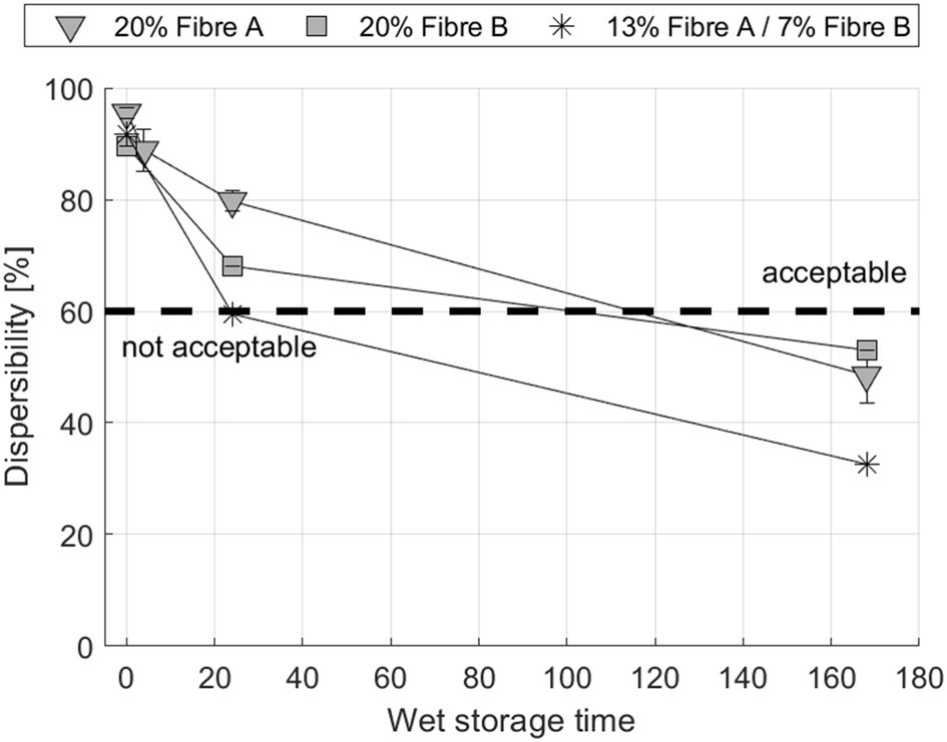
Reduction of wet wipe dispersibility due to storage in water for wipes from industrial pilot production. The same bleached kraft pulp was used in all samples and the viscose fibre was altered. All wet wipes from industrial pilot production show a considerable decline in dispersibility over storage time. Error bars represent one standard deviation.

**Figure 5:**
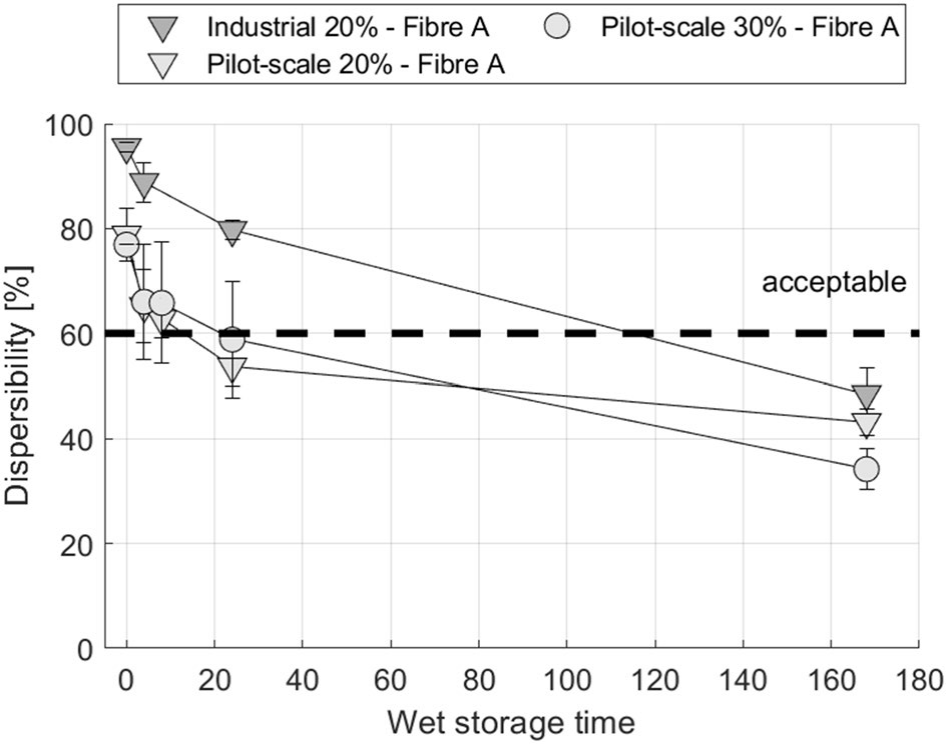
Wet wipe dispersibility after storage in water. Wipes were made in laboratory pilot-scale production (cf. Table 2 P.AB.20, P.AB.30) and industrial pilot production (cf. Table 3 I.AB.20), using the same fibres. All products show the same dispersibility ageing (deterioration in dispersibility). Error bars represent one standard deviation.

This solidification during the wet storage we call the ‘dispersibility ageing effect’. According to the INDA/EDANA guideline for wet wipes a minimum dispersibility of 60% is required^15^. The area below the dashed line at 60% dispersibility in Figs. 2, 3, 4 and 5 indicates insufficient dispersibility after being flushed. After two weeks of storage the wet wipes investigated in Fig. 2 hardly disintegrate at all in the slosh box test, their dispersibility is below 10%. This is only an effect of the wet storage, the initial dispersibility of the dry fabrics is well above 70%.

The wet storage time in our tests is even lower than the time commercial wet wipes are stored in their package before being used by consumers. The results from Fig. 2 are explaining the poor dispersibility of commercially available wet wipes observed in other work, even for grades labelled flushable^17,30^, as flushability tests are normally performed on the dry wipes before lotioning and wet storage. A slosh box flushability test that better reflects the real situation thus should be performed with wet wipes after a defined wet storage time, or should even test commercially available wet wipes collected from retailers.

Figure 3 presents the results for the wet wipes produced on the laboratory pilot plant for different types of viscose fibres and wood pulps. Except for the wipe with unbleached pulp, all wet wipes show a similar decline in dispersibility over the wet storage time. Although there is an offset in the level of the curves the slope is similar. All of the tested wipes have better dispersibility in the beginning, with an ongoing loss of this property over the time. The dispersibility ageing effect was here found for most variations of viscose fibres and wood pulps. This is in good accordance to findings in other publications^17,30,43^ where a large amount of wet wipes with different materials was tested and none are reaching satisfactory dispersibility. Most wipes show a considerable decline in dispersibility, only the wipe produced with unbleached pulp remains well dispersible over the entire storage time. In our investigations, Fig. 3, only one viscose-pulp combination was able to overcome the loss in dispersibility. It was the wipes produced from unbleached softwood kraft pulp and band-shaped viscose fibres, validating our fourth research hypothesis.

Deterioration in dispersibility leads to accumulations of wet wipes or their fragments in various parts of the sewer system where small agglomerations can cause clogging at sewer pumps^26^ which seem to be the main point of accumulation of wet wipes. A dispersibility of about 60% after 24 h (compare e.g. Fig. 3, bleached pulp and 30% viscose fibre A) means that a wipe decomposes to only smaller pieces whereas a dispersibility of 10% and below (e.g. Bleached pulp and 30% viscose fibre B at 168 h) means that the tested wipe hardly disperses at all, apart from small fragments. In any case, also small pieces and fibres are able to attribute to pump cloggings^27^, however large pieces are much more critical. Typical supply chains exceed 168 h storage by far, leading to wet storage times of several weeks for consumer wet wipes. Considering the severely reduced dispersibility after only 168 h of wet storage we can assume that these wet wipes may not be able to disperse after consumer disposal, even when they show excellent dispersibility directly after production.

Figure 3 also demonstrates that the type of viscose fibre is playing a role for dispersibility. Utilizing the same pulp, viscose fibre A, which has a flat cross section, provides better dispersibility than viscose fibre B with a circular cross section. This has also been observed earlier^37^, there the improved dispersibility had been attributed to the better access of shear forces to the flat viscose fibres during disintegration.

Similar to the findings shown in Fig. 3 tests were carried out for wipes from industrial pilot scale production, manufactured by a commercial producer of nonwovens for wet wipes. Figure 4 depicts the results for wipes using the same viscose fibres and a similar bleached pulp as in the laboratory pilot-scale trials. I.AB.20 for example represents a wipe with flat viscose fibres (Fibre A). Again, the wipes show good dispersibility when tested dry but they undergo a loss in dispersibility over the wet storage time. This demonstrates that the reduction of dispersibility during wet storage occurs for both, wipes produced at industrial pilot scale as well as laboratory pilot scale. A direct comparison is shown in Fig. 5. Although there are differences in the starting level, both, industrial and laboratory pilot-scale production wet wipes, show a similar rate of decline in dispersibility which is supporting the second statement of our working hypothesis. Considering that there are differences in machinery and parameters between laboratory and industrial pilot scale production it is reasonable to conclude that the time-dependent loss in dispersibility is rooted in the fibres used, and not in the production process. On the other hand, the slightly enhanced dispersibility of the industrial wipes could origin in the in-line production process and single drying of this method. However, the dispersibility reaches the same level after 168 h showing the good agreement of the methods according to investigations on the loss in dispersibility during wet storage.

In our work we were able to demonstrate that dispersibility ageing is visible in a huge variety of material blends and occurs both in deionized water and water-based lotion. At this point we cannot provide a conclusive explanation for this phenomenon. Based on literature research we are, however, able to discuss some hypotheses. Changing the used short fibre component, i.e. the wood pulp from a bleached to an unbleached grade, showed to strongly reduce the loss in dispersibility. Wood pulp fibres take up water causing them to swell, which increases the surface area of the fibres and reduces their stiffness. Findings from composite material science suggest that swelling of cellulosic materials continues for up to 168 h^44,45^ or even longer^46^. Fibres in a wet wipe are entangled with each other building different types of ribbons and knots^33,37^. Swelling increases the dimensions of the fibre and reduces fibre stiffness which could cause the described ribbons and knots to tighten and therefore enhancing the strength of the wet wipe. Also, surface phenomena such as interdiffusion, where the cellulose molecules of adjacent fibres penetrate each other^47^, are increasing network adhesion and thus the resistance against disintegration. For clarification of the mechanisms future research should focus on the change of the fibre material characteristics inflicted by the wet storage. The reported differences between usage of bleached and unbleached pulp are a good starting point, as hemicelluloses (which are removed during bleaching processes) are able to increase the ability of pulps to take up water when the pulp is dried. Also, the fibre surface is affected by bleaching which could impact interdiffusion effects.

## Conclusions

It has been shown consistently that storage of wet wipes in water or water-based lotion, as it is the case for commercial products, is severely reducing their ability to disintegrate properly after usage. After 24 h, latest after 168 h, the dispersive properties for most of the tested hydroentangled wetlaid wipes are strongly reduced. Dispersibility of wipes tested without wet storage, represented as 0-h dispersibilities in Figs. 3, 4 and 5, are in good accordance to recent publications^11,32,33,37^, confirming that initially the wet wipes are disintegrating well. The problem clearly is the storage of the wipes in water or lotion. It is not very intuitive that storage of a wet wipe fabric in water (or a water-based liquid) reduces its ability to be dispersed in water, yet this is exactly what is happening. Possible mechanisms causing this dispersibility ageing effect could be related to long term swelling processes^44–46^, which are common to cellulosic fibres, swelling mediated interdiffusion between the fibre surfaces or mechanical deformation of the softened fibre networks during wet storage.

The reason that this effect has not been described, is probably due to the fact that this behaviour is quite unexpected and thus has not been examined so far. Nevertheless, it has profound environmental implications. In the supply chain, the time between producing a wet wipe (i.e. putting it in wet storage in the consumer package) and the sale in stores is by far longer than 168 h. Monitoring dispersibility of wet wipes after shelf storage can help to reduce sewer blockage and ensure proper dispersibility and faster biodegradability of the wipes.

As one key consequence of the work presented here, we are suggesting to adapt the standard testing procedures for wet wipe flushability in such a way that also a possible reduction of dispersibility over time due to dispersibility ageing is covered. Such a procedure needs to include the test of wipes also after several weeks, in order to correctly reflect the end use of the commercial product.

The dispersibility ageing effect has been observed equivalently for industrial-pilot and laboratory pilot-scale production wet wipes, the decrease in dispersibility is similar. For pilot-scale produced wipes, we demonstrated that using a combination of unbleached softwood pulp and band shaped viscose fibres, wet wipes with good dispersibility properties over wet storage can be made, see Fig. 3. This shows that using appropriate fibres, truly flushable and biodegradable wet wipes from purely lignocellulosic material can be produced.

## Supplementary Information

Supplementary Information.

## References

[1] Mango, P. The future of global nonwoven wipes market forecasts to 2023. In *57th Dornbirn Global Fiber Congress* vol. 44 (2018).

[2] Mango P (2004). Flushable wipes past present future. Nonwovens Ind..

[3] Blackburn RS (2005). Biodegradable and Sustainable Fibres.

[4] Polman EMN, Gruter GJM, Parsons JR, Tietema A (2021). Comparison of the aerobic biodegradation of biopolymers and the corresponding bioplastics: A review. Sci. Total Environ..

[5] Okada M (2002). Chemical syntheses of biodegradable polymers. Prog. Polym. Sci..

[6] Narancic T (2018). Biodegradable plastic blends create new possibilities for end-of-life management of plastics but they are not a panacea for plastic pollution. Environ. Sci. Technol..

[7] Zambrano MC (2019). Microfibers generated from the laundering of cotton, rayon and polyester based fabrics and their aquatic biodegradation. Mar. Pollut. Bull..

[8] Pantoja Munoz L, Gonzalez Baez A, McKinney D, Garelick H (2018). Characterisation of “flushable” and “non-flushable” commercial wet wipes using microRaman, FTIR spectroscopy and fluorescence microscopy: To flush or not to flush. Environ. Sci. Pollut. Res..

[9] Ó Briain O, MarquesMendes AR, McCarron S, Healy MG, Morrison L (2020). The role of wet wipes and sanitary towels as a source of white microplastic fibres in the marine environment. Water Res..

[10] Eren B, Karadagli F (2012). Physical disintegration of toilet papers in wastewater systems: Experimental analysis and mathematical modeling. Environ. Sci. Technol..

[11] Zhang Y (2019). A new dispersible moist wipe from wetlaid/spunlace nonwoven: Development and characterization. J. Ind. Text..

[12] Soukupova V, Boguslavsky L, Anandjiwala RD (2007). Studies on the properties of biodegradable wipes made by the hydroentanglement bonding technique. Text. Res. J..

[13] Zhang Y, Deng C, Qu B, Zhan Q, Jin X (2017). A study on wet and dry tensile properties of wood pulp/lyocell wetlace nonwovens. IOP Conf. Ser. Mater. Sci. Eng..

[14] Mao N, Russell SJ (2006). A framework for determining the bonding intensity in hydroentangled nonwoven fabrics. Compos. Sci. Technol..

[15] INDA & EDANA. *Guidelines for Assessing the Flushability of Disposable Nonwoven Products A Process for Assessing the Compatibility of Disposable Nonwoven Products with Plumbing and Wastewater* (2018).

[16] IWSFG. *International Water Services Flushability Group Flushability Specifications*. https://www.iwsfg.org/iwsfg-flushability-specification/ (2018).

[17] Joksimovic D, Khan A, Orr B (2020). Inappropriate disposal of ‘flushable’ consumer products—reasons for concern. Water Sci. Technol..

[18] Atasagun H, Bhat G (2018). Advancement in flushable wipes: Modern technologies and characterization. J. Ind. Text..

[19] Campbell, E. No ‘flushable’ wet wipes tested so far pass water industry tests. *BBC* https://www.bbc.com/news/uk-46188354 (2018).

[20] Kary T (2019). Fatberg Fight NYC Goes to War Against Flushable Wipes.

[21] Hassan, J. Britain’s latest ‘fatberg,’ a mass of grease and wet wipes in a sewer, is longer than 6 double-decker buses. *The Washington Post*. https://www.washingtonpost.com/world/2019/01/09/britains-latest-fatberg-mass-grease-wet-wipes-is-longer-than-double-decker-buses/?utm_term=.1c37fa8d2316 (2019).

[22] Flegenheimer M (2015). Wet wipes box says flush. New York’s Sewer System Says Don’t. N.Y. Times.

[23] Taylor M (2017). Total Monster’: Fatberg Blocks London Sewage System.

[24] He X (2011). Evidence for fat, oil, and grease (FOG) deposit formation mechanisms in sewer lines. Environ. Sci. Technol..

[25] Kusum SA, Pour-Ghaz M, Ducoste JJ (2020). Reducing fat, oil, and grease (FOG) deposits formation and adhesion on sewer collection system structures through the use of fly ash replaced cement-based materials. Water Res..

[26] Thamsen PU, Gunkel M, Waschnewski J, Mitchell R-L (2017). Investigations into wastewater composition focusing on nonwoven wet wipes. Czas. Tech..

[27] Mitchell RL, Gunkel M, Waschnewski J, Thamsen PU (2020). Nonwoven wet wipes can be hazardous substances in wastewater systems—evidences from a field measurement campaign in Berlin, Germany. Front. Water Energy Nexus Nat. Based Solut. Adv. Technol. Best Pract. Environ. Sustain..

[28] Okamoto, N. *Case Study of Preventing Clogging of Pumps Caused by Nonwoven Wipes in Tokyo*. 1812–1818 (2018).

[29] Karadagli F, Theofanidis F, Eren B (2021). Consumers’ evaluation of flushable products with respect to post-disposal effects in wastewater infrastructures. J. Clean. Prod..

[30] Atasağun HG, Bhat GS (2019). Assessing the structural, mechanical and dispersible characteristics of flushable nonwovens. Text. Res. J..

[31] Khan, A., Orr, B. & Joksimovic, D. *Defining “Flushability” for Sewer Use*. https://www.ryerson.ca/content/dam/water/Research/FinalReport-FlushablesApril1.pdf (2019).

[32] Zhang Y, Zhao Y, Latifi M, Wang R, Jin X (2018). Investigation of the mechanical and dispersible properties of wood pulp/Danufil wetlaid nonwovens with/without hydroentanglement. J. Text. Inst..

[33] Zhang Y, Jin X (2018). The influence of pressure sum, fiber blend ratio, and basis weight on wet strength and dispersibility of wood pulp/Lyocell wetlaid/spunlace nonwovens. J. Wood Sci..

[34] Karadagli F, McAvoy DC, Rittmann BE (2009). Development of a mathematical model for physical disintegration of flushable consumer products in wastewater systems. Water Environ. Res..

[35] Tang Y, Jin XY (2012). Study on flushability testing of wood pulp composite spunlaced nonwovens. Adv. Mater. Res..

[36] Deng C (2018). Environmentally friendly and breathable wet-laid hydroentangled nonwovens for personal hygiene care with excellent water absorbency and flushability. R. Soc. Open Sci..

[37] Zhang Y, Xu Y, Zhao Y, Huang C, Jin X (2019). Effects of short-cut fiber type and water-jet pressure sum on wet strength and dispersibility of wood pulp-based wetlaid/spunlace wipes. Eur. J. Wood Wood Prod..

[38] Drinkwater, A. & Moy, F. Wipes in sewer blockage study. *21st Century Drain. Program.* 13 (2017).

[39] Cook GJ (1984). Handbook of Textile Fibres: Man-Made Fibres.

[40] Wilkes AG, Woodings C (2001). The viscose process. Regenerated Cellulose Fibers.

[41] Marsh, R. G. *Stable Lotion Emulsion Composition and Wet Wipes*. (2016).

[42] Tipper MJ (2016). Flushability of Nonwoven Wet Wipes.

[43] Durukan S, Karadagli F (2019). Physical characteristics, fiber compositions, and tensile properties of nonwoven wipes and toilet papers in relevance to what is flushable. Sci. Total Environ..

[44] Fang H, Zhang Y, Deng J, Rodrigue D (2013). Effect of fiber treatment on the water absorption and mechanical properties of hemp fiber/polyethylene composites. J. Appl. Polym. Sci..

[45] Jawaid M, Abdul Khalil HPS, Noorunnisa Khanam P, Abu Bakar A (2011). Hybrid composites made from oil palm empty fruit bunches/jute fibres: Water absorption, thickness swelling and density behaviours. J. Polym. Environ..

[46] Tajvidi M, Najafi SK, Moteei N (2006). Long-term water uptake behavior of natural fiber/polypropylene composites. J. Appl. Polym. Sci..

[47] Hirn U, Schennach R (2015). Comprehensive analysis of individual pulp fiber bonds quantifies the mechanisms of fiber bonding in paper. Sci. Rep..

