# Author Correction: Reduced dispersibility of flushable wet wipes after wet storage

**DOI:** 10.1038/s41598-021-94248-8

**Published:** 2021-07-26

**Authors:** Thomas Harter, Ingo Bernt, Stefanie Winkler, Ulrich Hirn

**Affiliations:** 1 Institute of Bioproducts and Paper Technology, Graz University of Technology, Inffeldgasse 23, 8010 Graz, Austria; 2 Kelheim Fibres GmbH, Regensburger Straße 109, Kelheim, Germany; 3 CD Laboratory for Fibre Swelling and Paper Performance, Inffeldgasse 23, 8010 Graz, Austria

Correction to: *Scientific Reports* 10.1038/s41598-021-86971-z, published online 12 April 2021

The original version of this Article contained errors.

The official method to test wet wipe flushability in the USA is the guideline INDA/EDANA GD4 (2018) 'Guidelines for Assessing the Flushability of Disposable Nonwoven Products'. We now clarified that the measurement method to assess flushability utilized in the article deviates from INDA/EDANA GD4 in several major aspects, as described in the new Supplementary File. Therefore, from the change in dispersibility found in the publication no conclusion can be drawn for the dispersibility of commercial wet wipes.

The Article has been updated to reflect these clarifications. In the Abstract,

“Loss of dispersibility is found for both, wet wipes from industrial production and wipes produced on pilot facilities.”

now reads:

“Loss of dispersibility is found for both, wet wipes from industrial pilot production and wipes produced on laboratory pilot facilities.”

In the Introduction,

“This loss of dispersibility is demonstrated for wipes from industrial production as well as for wipes produced on pilot scale. We will also demonstrate that by selecting suitable fibres to produce the wet wipe, it is possible to obtain wipes with little to no dispersibility ageing, thus proving that the widespread wetlaid/hydroentanglement process is suited to manufacture biodegradable and truly flushable wet wipes. Still, stable dispersibility over wet storage was only found for one set of fibres not currently used for commercial products as far as we know, indicating that typical commercially available wet wipes are deteriorating in their dispersibility properties during wet storage in the consumer package.”

now reads:

“This loss of dispersibility is demonstrated for wipes from industrial pilot production as well as for wipes produced on laboratory pilot scale. We will also demonstrate that by selecting suitable fibres to produce the wet wipe, it is possible to obtain wipes with little to no dispersibility ageing, thus proving that the widespread wetlaid/hydroentanglement process is suited to manufacture biodegradable and truly flushable wet wipes. Still, stable dispersibility over wet storage was only found for one set of fibres not currently used for commercial products as far as we know, indicating that typical commercially available wet wipes are deteriorating in their dispersibility properties during wet storage in the consumer package, at least in the first 168 hours.”

In Material and Methods, subheading ‘Wet wipe production—pilot scale’ now reads ‘Wet wipe production—laboratory pilot scale’. In this section,

“The pilot-scale method has been adapted to mimic the industrial production process described below.”

now reads:

“The laboratory pilot-scale method has been adapted to mimic the industrial pilot production process described below.”

“P.AB.20 therefore refers to a wipe that is produced with the pilot-scale method using viscose fibre A (A) and bleached softwood kraft pulp (B).”

now reads:

“P.AB.20 therefore refers to a wipe that is produced with the laboratory pilot-scale method using viscose fibre A (A) and bleached softwood kraft pulp (B).”

In Material and Methods, subheading ‘Wet wipe production—industrial scale’ now reads ‘Wet wipe production—industrial pilot scale’. In this section,

“Both components, viscose fibres and pulp, were the same products like in the pilot-scale produced fabrics, but from different production lots. The industrial machinery to produce the fabrics was similar to the pilot-scale method. Web forming was achieved using the wetlaid process to build the initial network of the nonwoven. The hydroentanglement process in the industrial scale is carried out in-line with the web forming.”

now reads:

“Both components, viscose fibres and pulp, were the same products like in the laboratory pilot-scale produced fabrics, but from different production lots. The industrial machinery to produce the fabrics was similar to the pilot-scale method. Web forming was achieved using the wetlaid process to build the initial network of the nonwoven. The hydroentanglement process in the industrial pilot scale is carried out in-line with the web forming.”

In Material and Methods, under the subheading ‘Dispersibility measurement—slosh box disintegration test’,

“The test specifications used in this work were the same as provided by the INDA/EDANA guidelines^15^”

now reads:

“The test specifications used in this work were, with alterations as listed in Supplementary File, similar to those provided by the INDA/EDANA guidelines^15^”

In Results and discussion,

“Figure 3 presents the results for the wet wipes produced on the pilot plant for different types of viscose fibres and wood pulps.”

now reads:

“Figure 3 presents the results for the wet wipes produced on the laboratory pilot plant for different types of viscose fibres and wood pulps.”

“Considering the severely reduced dispersibility after only 168 h of wet storage we can assume that these wet wipes are not able to disperse after consumer disposal, even when they show excellent dispersibility directly after production.”

now reads:

“Considering the severely reduced dispersibility after only 168 h of wet storage we can assume that these wet wipes may not be able to disperse after consumer disposal, even when they show excellent dispersibility directly after production.”

Furthermore, in the Results and discussion,

“Similar to the findings shown in Fig. 3 tests were carried out for wipes from industrial scale production, manufactured by a commercial producer of nonwovens for wet wipes. Figure 4 depicts the results for wipes using the same viscose fibres and a similar bleached pulp as in the pilot-scale trials. I.AB.20 for example represents a wipe with flat viscose fibres (Fibre A). Again, the wipes show good dispersibility when tested dry but they undergo a loss in dispersibility over the wet storage time. This demonstrates that the reduction of dispersibility during wet storage occurs for both, wipes produced at industrial scale as well as pilot scale. A direct comparison is shown in Fig. 5. Although there are differences in the starting level, both, industrial and pilot-scale production wet wipes, show a similar rate of decline in dispersibility which is supporting the second statement of our working hypothesis. Considering that there are differences in machinery and parameters between pilot- and industrial scale production it is reasonable to conclude that the time-dependent loss in dispersibility is rooted in the fibres used, and not in the production process.”

now reads:

“Similar to the findings shown in Fig. 3 tests were carried out for wipes from industrial pilot scale production, manufactured by a commercial producer of nonwovens for wet wipes. Figure 4 depicts the results for wipes using the same viscose fibres and a similar bleached pulp as in the laboratory pilot-scale trials. I.AB.20 for example represents a wipe with flat viscose fibres (Fibre A). Again, the wipes show good dispersibility when tested dry but they undergo a loss in dispersibility over the wet storage time. This demonstrates that the reduction of dispersibility during wet storage occurs for both, wipes produced at industrial pilot scale as well as laboratory pilot scale. A direct comparison is shown in Fig. 5. Although there are differences in the starting level, both, industrial and laboratory pilot-scale production wet wipes, show a similar rate of decline in dispersibility which is supporting the second statement of our working hypothesis. Considering that there are differences in machinery and parameters between laboratory and industrial pilot scale production it is reasonable to conclude that the time-dependent loss in dispersibility is rooted in the fibres used, and not in the production process.”

Finally, in the Conclusions,

“In the supply chain, the time between producing a wet wipe (i.e. putting it in wet storage in the consumer package) and the sale in stores is by far longer than 168 h. Thus, an even more severe loss in dispersibility may take place for wet wipes in commercial end use. Monitoring dispersibility of wet wipes after shelf storage will help to reduce sewer blockage and ensure proper dispersibility and faster biodegradability of the wipes.”

now reads:

“In the supply chain, the time between producing a wet wipe (i.e. putting it in wet storage in the consumer package) and the sale in stores is by far longer than 168 h. Monitoring dispersibility of wet wipes after shelf storage can help to reduce sewer blockage and ensure proper dispersibility and faster biodegradability of the wipes.”

“The dispersibility ageing effect has been observed equivalently for industrial- and pilot-scale production wet wipes, the decrease in dispersibility is similar.”

now reads:

“The dispersibility ageing effect has been observed equivalently for industrial pilot and laboratory pilot scale production wet wipes, the decrease in dispersibility is similar.”

Additionally, the legends of Figure 4 and 5 have been updated. In the legend of Figure 4,

“Reduction of wet wipe dispersibility due to storage in water for wipes from industrial production. The same bleached kraft pulp was used in all samples and the viscose fibre was altered. All commercial products show a considerable decline in dispersibility over storage time. Error bars represent one standard deviation.”

now reads:

“Reduction of wet wipe dispersibility due to storage in water for wipes from industrial pilot production. The same bleached kraft pulp was used in all samples and the viscose fibre was altered. All wet wipes from industrial pilot production show a considerable decline in dispersibility over storage time. Error bars represent one standard deviation.”

In the legend of Figure 5,

“Wipes were made in pilot-scale production (cf. Table 2 P.AB.20, P.AB.30) and industrial production (cf. Table 3 I.AB.20), using the same fibres.”

now reads:

“Wipes were made in laboratory pilot-scale production (cf. Table 2 P.AB.20, P.AB.30) and industrial pilot production (cf. Table 3 I.AB.20), using the same fibres.”

The legends of Tables 2 and 3 have also been updated. In the legend of Table 2,

“Table 2. List of produced pilot-scale nonwovens. PW stands for pilot web, VA/VB for viscose type and BSK/UBSK for the pulp grade.”

now reads:

“Table 2. List of produced laboratory pilot-scale nonwovens. PW stands for pilot web, VA/VB for viscose type and BSK/UBSK for the pulp grade.”

In the legend of Table 3,

“Table 3. List of nonwovens from industrial production. IW stands for industrial web, VA/VB for viscose type and BSK for bleached softwood kraft pulp.”

now reads:

“Table 3. List of nonwovens from industrial pilot production. IW stands for industrial web, VA/VB for viscose type and BSK for bleached softwood kraft pulp.”

The original Article has been corrected, and is now accompanied by a new Supplementary Information file.

