# Supplementary material for: Reduced dispersibility of flushable wet wipes after wet storage
Source: Sci Rep. 2021 Apr 12;11:7942. doi: 10.1038/s41598-021-86971-z (PMC8042014; doi:10.1038/s41598-021-86971-z)
Supplement: Supplementary file 1 — Supplementary Information. [file 41598_2021_86971_MOESM1_ESM.docx]

Reduced dispersibility of flushable wet wipes after wet storage

Thomas Harter, Ingo Bernt, Stefanie Winkler & Ulrich Hirn

**Supplementary File**

Detailed description of the differences in flushability measurements between the article and INDA/EDANA GD4

The differences are summarized in Supplementary Table 1.

|  | **INDA EDANA GD 4** | **This study** |
| --- | --- | --- |
|  |  |  |
| **Evaluation replicants (each)** | 6 | 3 |
| **Test time in (min)** | 60 | 30 |
|  |  |  |
| **Test water temp (°C)** | 22° C (+/- 3° C) | 18 - 23°C |
|  |  |  |
| **Shower head flow rate (liter)** | 4 | Not applied |
| **Shower head spray nozzle distance (cm)** | 10 to 15 | Not applied |
| **Shower head spray time (min)** | 1 | 0 |

**Supplementary Table 1** Comparison between flushability guidelines INDA/EDANA Ed. 4 and the method used in our work.

One crucial difference also outlined in our study is the time wipes are agitated in the slosh box.

Reducing the disintegration time – as applied in the publication - provides less harsh conditions for the wipes leading to lower dispersibility values.

More importantly, in our measurements the largest change in dispersibility has been observed in the very first week after production. During this time the wipes are usually not sold as they need to be distributed to the retailer. Thus the results are different than from commercial wet wipes.

The recommended sample amount was set to 3 samples as the used apparatus allows 3 wipes to be tested simultaneously. Therefore, 3 samples at each wet storage time were tested at the exactly same condition. The INDA/EDANA guidelines suggest to measure 6 wipes to make significant statements about the flushability of wet wipes.

Another difference between the two test methods lies within the treatment after the remains of the slosh box are poured and collected on the 12.5 mm perforated sieve. The INDA/EDANA guidelines describe a shower-rinsing as described in their supplementary guidance documents SG004.R1(18). These treatments could also increase the dispersibility values as the rinsing of the sieve leads to more fibres and flakes passing the 12.5 mm sieve. This additional treatment was not used in our work resulting most probably in lower dispersibility values.

The test water temperature was not controlled as recommend in the guidelines. The recommended ratio, varying between 19°C and 25°C, is very broad. In our measurements tap water was stored in a container before being used. Therefore, the range of temperature for the water used in our measurements is likely to be close to room temperature at about 18°C – 23°C.
